# ROS-mediated plasmodesmal regulation requires a network of an Arabidopsis receptor-like kinase, calmodulin-like proteins, and callose synthases

**DOI:** 10.3389/fpls.2022.1107224

**Published:** 2023-01-19

**Authors:** Minh Huy Vu, Tae Kyung Hyun, Sungwha Bahk, Yeonhwa Jo, Ritesh Kumar, Dhineshkumar Thiruppathi, Arya Bagus Boedi Iswanto, Woo Sik Chung, Rahul Mahadev Shelake, Jae-Yean Kim

**Affiliations:** ^1^ Division of Applied Life Science (BK21 Four Program), Plant Molecular Biology and Biotechnology Research Center, Gyeongsang National University, Jinju, Republic of Korea; ^2^ Department of Industrial Plant Science and Technology, College of Agricultural, Life and Environmental Sciences, Chungbuk National University, Cheongju, Republic of Korea; ^3^ College of Biotechnology and Bioengineering, Sungkyunkwan University, Suwon, Republic of Korea; ^4^ Division of Life Science, Gyeongsang National University, Jinju, Republic of Korea; ^5^ Research and Development Center, Nulla Bio Inc 501 Jinju-daero, Jinju, Republic of Korea

**Keywords:** callose, plasmodesmata, ROS perception, abiotic and biotic stress, receptor-like kinase (RLK)

## Abstract

Plasmodesmata (PD) play a critical role in symplasmic communication, coordinating plant activities related to growth & development, and environmental stress responses. Most developmental and environmental stress signals induce reactive oxygen species (ROS)-mediated signaling in the apoplast that causes PD closure by callose deposition. Although the apoplastic ROS signals are primarily perceived at the plasma membrane (PM) by receptor-like kinases (RLKs), such components involved in PD regulation are not yet known. Here, we show that an Arabidopsis NOVEL CYS-RICH RECEPTOR KINASE (NCRK), a PD-localized protein, is required for plasmodesmal callose deposition in response to ROS stress. We identified the involvement of NCRK in callose accumulation at PD channels in either basal level or ROS-dependent manner. Loss-of-function mutant (*ncrk*) of NCRK induces impaired callose accumulation at the PD under the ROS stress resembling a phenotype of the PD-regulating *GLUCAN SYNTHASE-LIKE 4* (*gsl4*) knock-out plant. The overexpression of transgenic NCRK can complement the callose and the PD permeability phenotypes of *ncrk* mutants but not kinase-inactive NCRK variants or Cys-mutant NCRK, in which Cys residues were mutated in Cys-rich repeat ectodomain. Interestingly, NCRK mediates plasmodesmal permeability in mechanical injury-mediated signaling pathways regulated by GSL4. Furthermore, we show that NCRK interacts with calmodulin-like protein 41 (CML41) and GSL4 in response to ROS stress. Altogether, our data indicate that NCRK functions as an upstream regulator of PD callose accumulation in response to ROS-mediated stress signaling pathways.

## 1 Introduction

Biotic and abiotic stresses, such as pathogens, insects, drought, waterlogging, and heat or cold, augment and intensify the risks to food security and affect agricultural production (Lesk et al., 2016; Bailey-Serres et al., 2019). The increased frequency and intensity of various biotic and abiotic stresses highlight the need to understand the mechanisms that help plants to resist such stresses (Zandalinas et al., 2021; Shelake et al., 2022). Mechanical wounding caused by different stresses activates plant defense pathways similar to those triggered by insects and herbivores (León et al., 2001; Gatehouse, 2002; War et al., 2012; Savatin et al., 2014). Also, wounded plant parts are prone to nutrient loss and provide entry points to phytopathogens (Savatin et al., 2014). Reactive oxygen species (ROS) and calcium (Ca^2+^) waves play a crucial role in integrating different stress response signaling networks and activating the plant defense mechanisms. In addition to activating acclimation mechanisms in the plant tissues exposed to stress, various abiotic stresses, as well as mechanical injury, can trigger rapid systemic responses at the whole plant level (Kaya et al., 2014; Cui and Lee, 2016; Mangano et al., 2017; Toyota et al., 2018; Wu et al., 2018; Cheval et al., 2020), resulting in the development of stress memory that protects the plants from subsequent exposures to the same stress.

Controlling the cell-to-cell movement *via* callose-mediated plasmodesmal regulation is one of the prevalent modes of action adopted by the plant immune system during stress (Iswanto et al., 2021). Callose, a β-1,3-glucan, is synthesized and degraded dynamically at the plasmodesmata (PD) neck, which modulates PD pore size and regulates the diffusion of molecules and signals (Kumar et al., 2015; Amsbury et al., 2018; Wu et al., 2018). ROS and callose were proposed to be linked through the function of the GLUCAN SYNTHASE-LIKE 4 (GSL4)/callose synthase 8 (CalS8) during wound stress in Arabidopsis (*Arabidopsis thaliana*) (Cui and Lee, 2016). Hydrogen peroxide (H_2_O_2_) is a major ROS species involved in plant signaling pathways due to its unique aspects, such as rapid movability across the plasma membrane (PM), the relatively long half-life, oxidizes proteins, and possess properties similar to water (Castro et al., 2021). H_2_O_2_ activates the signaling through a cascade of proteins and is implicated in plant adaptation to developmental and stress responses (Mittler et al., 2022). Extracellular H_2_O_2_ (eH_2_O_2_) at the apoplast is sensed by receptors and transduced to the cytosol. It then enables alterations in different protein structures, kinases/phosphatase molecular switches, localization, and protein-protein interactions (Castro et al., 2021). A primary mechanism for H_2_O_2_ sensing is the oxidative modification of Cysteine (Cys) residues, in which the availability of different oxidation states results in a diverse range of post-translational modifications (Waszczak et al., 2015). Recently, the apoplast ROS sensor named HYDROGEN-PEROXIDE-INDUCED CALCIUM INCREASES 1 (HPCA1) perceives the H_2_O_2_ molecule by modifying the conformation of the extracellular domain (ED), also called hydrogen peroxide (HP) domain (Wu et al., 2020). The HP domain, which contains four Cys residues, is activated by eH_2_O_2_, increasing the kinase activity and resulting in induced Ca^2+^ influx into the cytosol (Wu et al., 2020). However, HPCA1 is essential for local and systemic cell-to-cell ROS signaling in the local response to bacterial and salinity stress treatments, but not wounding, suggesting that other extracellular ROS receptors exist at PD that mediate the ROS-induced wounding stress response (Fichman et al., 2022).

Over a decade, many PD-related receptor proteins have been shown to regulate cell-to-cell movement *via* controlling the PD aperture during stress and development, such as PD-LOCALIZED PROTEIN 1 and 5 (PDLP1, PDLP5), QIĀN SHŎU KINASE (QSK1), CYS-RICH RECEPTOR-LIKE KINASE 2 (CRK2), and several members of PM-located Leucine-rich-repeat receptor-like kinases (RLKs) family (Lee et al., 2011; Grison et al., 2019; Hunter et al., 2019; Kimura et al., 2020; Fichman et al., 2021). Nevertheless, whether PD localized-receptor proteins are activated by eH_2_O_2_ that may participate in PD regulation by inducing callose deposition during wounding stress are currently unknown. ROS, Ca^2+^, phosphorylation, and electric signaling are rendered at the local wounding site to trigger rapid plant response (Sager and Lee, 2014; Toyota et al., 2018; Vega-Muñoz et al., 2020; Fichman et al., 2021; Shin et al., 2022). Previous studies have shown that changes in cytosolic Ca^2+^ signals regulate plasmodesmal flux and callose deposition following biotic and abiotic stresses (Leba et al., 2012; Wu et al., 2020). Calmodulin and Calmodulin-like (CML) proteins, Ca^2+^-dependent protein kinases (CDPKs), and calcineurin-B-like proteins are the major families of Ca^2+^-binding proteins in plants (Zeng et al., 2015; Yip Delormel and Boudsocq, 2019). CML9 negatively controls callose deposition after *Pseudomonas syringae pv. tomato* (Pto) DC3000 strain treatment in Arabidopsis (Leba et al., 2012). The PD-localized CML41 positively regulates the PD callose upon the flagellin epitope flg22 pathogen perception (Xu et al., 2017). Overall, several PD-located receptor and CML protein family members have been recently identified; but none have been characterized for their interacting partner proteins in the plasmodesmal regulation during the stress response.

Here, we characterize an Arabidopsis NOVEL CYS-RICH RECEPTOR KINASE (NCRK; AT2G28250), which acts as a key ROS receptor and is involved in callose-mediated PD regulation in response to external ROS treatment and wounding stress. Moreover, we characterize that NCRK is necessary for basal callose deposition and ROS-dependent PD closure, and these processes are mediated by its interactions with CML41 and GSL4. In addition, our study reveals that NCRK phosphorylation is essential for the ROS-mediated downstream signaling pathways during this process. We further characterize the function of the Cys-rich motif positioned on ED of NCRK (NCRK-ED) in ROS perception in response to eH_2_O_2_ treatment. Our findings indicate that NCRK plays a crucial role in the apoplast ROS signaling, linking the callose accumulation that requires the plant to acclimate to biotic and abiotic stresses.

## 2 Results

### 2.1 NCRK is a PD-localized RLK and controls basal callose deposition at the PD

**Figure 1:**
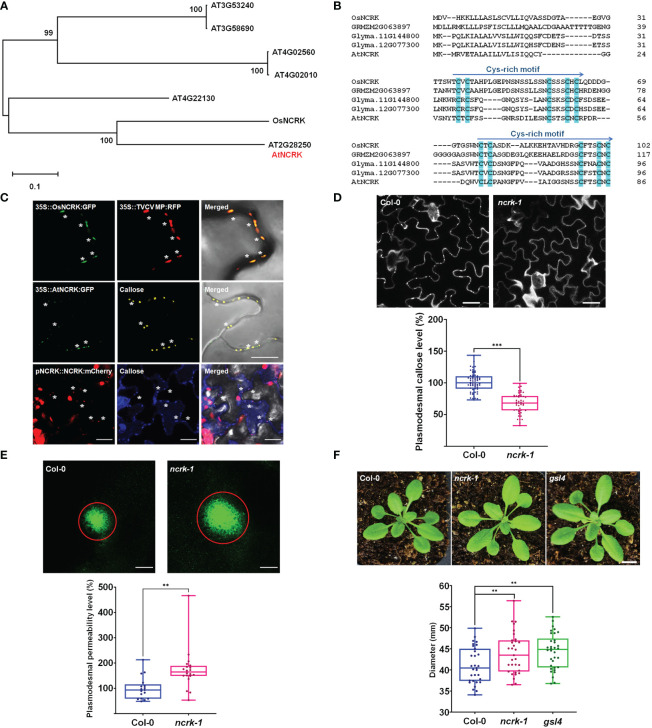
NCRK is a plasmodesmata-localized RLK that regulates basal PD permeability. **(A)** Phylogenetic tree of NCRK across species. **(B)** Conserved cysteine-rich repeat domain homolog sequence across species. **(C)** OsNCRK (upper panels) and NCRK (middle and bottom panels) co-localize with PD markers during transient expression, and NCRK (bottom panels) co-localizes with callose in the transgenic plant. **(D)** PD callose staining and its quantification in the leaf epidermal cells of wild-type Col-0 and *ncrk-1* (n≥12). Scale bar: 20 μm. **(E)** DANS assays on Col-0 and *ncrk-1*. The diameter of the fluorescent circle shows the level of cell-to-cell permeability of symplasmic dye (n≥12). Scale bar: 200 μm. **(F)** Plant images and quantification of rosette leaf diameter, Col-0 (n=31), *ncrk-1* (n=31), *gsl4* (n=34). Scale bar: 1 cm. The data are summarized in box plots in which the line within the box marks the median, the box signifies the upper and lower quartiles. The whiskers go down to the smallest value and up to the largest. Data were analyzed by a Student’s *t*-test. ns, not significant. ^**^ *P <*0.01, ^***^ *P* < 0.001.

Our previous study on rice PD-proteome analysis identified several members of the RLK family resided in the PD region, including an unpublished Os02g20400 (Jo, 2011; Jo et al., 2011). By using bioinformatic analysis, we identified an Arabidopsis RLK protein family member (AT2G28250, named NCRK) with the highest identity compared with Os02g20400 (named OsNCRK) (**Figure 1A**). This protein was formerly reported as an NCRK, a member of the RLK family with an unknown function (**Figure 1B**) (Molendijk et al., 2008). In Arabidopsis, NCRK could be hyperphosphorylated and partially co-localized with the small GTPase ARABIDOPSIS RAB HOMOLOG F2A (RapF2a) in endosomes (Molendijk et al., 2008). The amino acid alignment between RLK orthologs reveals that the extracellular receptor domain encoded by NCRK protein contains two repeats of about 40 amino acids sharing a unique and highly conserved Cys-rich motif, WXCXCX13–18CX3CXC across the species, suggesting their specific function (**Figure 1B**).

First, we examined the subcellular localization of OsNCRK and NCRK in the transient *Nicotiana benthamiana* plant by transiently expressing green fluorescent protein (GFP) fusion to the C-terminal of both proteins, followed by fluorescence imaging using confocal microscopy. OsNCRK and Arabidopsis NCRK showed punctate spots at the cell periphery; a typical pattern was observed resembling PD localization. To confirm if the spots are co-localized with PD, we co-expressed NCRK-GFP with PD markers such as *Turnip vein-clearing virus* (*TVCV*) movement protein, callose staining with Aniline Blue dye, PDLP1 and PDLP5 (Thomas et al., 2008; Jo et al., 2011; Lee et al., 2011). NCRK-GFP fluorescence spots co-localized well with the four PD markers (**Figure 1C** and **Supplementary Figure 1A**). To quantify the percentage of NCRK co-localization at the PD area, we measured the PD index by calculating the ratio of mean fluorescent intensity of the PD enrichment area and its PM neighbor area (**Supplementary Figure 1C**). NCRK was highly localized in the PD area, similarly with PDLP1 or PDLP5, but not with RECEPTOR DEAD KINASE1 (RDK1) used as PM markers (Kumar et al., 2017) (**Supplementary Figure 1B**). Analyses of transgenic seedlings expressing a *GUS* gene driven by Arabidopsis *NCRK* promoter (a) showed that the underlying gene (*gus*) is broadly expressed throughout the whole seedling and more prominently in the root, hypocotyl, and cotyledon. In cotyledon, relatively strong expression was observed in vascular tissues but to less extent in mesophyll cells (**Supplementary Figure 2B**). Analyses of *pNCRK::NCRK-mCherry* transgenic lines showed that the fluorescence was co-localized or localized to the vicinity of the aniline blue-stained callose (**Figure 1D**). To investigate the potential function of NCRK in PD regulation, we obtained T-DNA insertion lines (*ncrk-1, ncrk-2*) (**Supplementary Figure 2A**) and performed the PD callose staining experiment. *ncrk-1 and ncrk-2* mutants showed less basal callose accumulation (**Figure 1D** and **Supplementary Figure 3C**). *ncrk-1* mutant plants exhibited increased PD permeability and decreased PD callose compared with wild-type plants in the leave and hypocotyl system (**Figure 1E** and **Supplementary Figures 3A, B**), suggesting the role of NCRK in callose deposition and PD permeability regulation. The callose turnover of the plant showed defective growth and development or was even lethal except for some mutant genes such as *gsl4*. Interestingly, the *ncrk-1* mutant plant displayed enhanced developmental growth compared with the wild-type, similar to the *gsl4* mutant plant, showing a reduced PD callose level and increased PD permeability (**Figure 1F**). These data indicate that NCRK is a plasmodesmal RLK protein family member, which controls basal callose accumulation and PD permeability.

### 2.2 NCRK is involved in ROS-induced callose deposition

**Figure 2:**
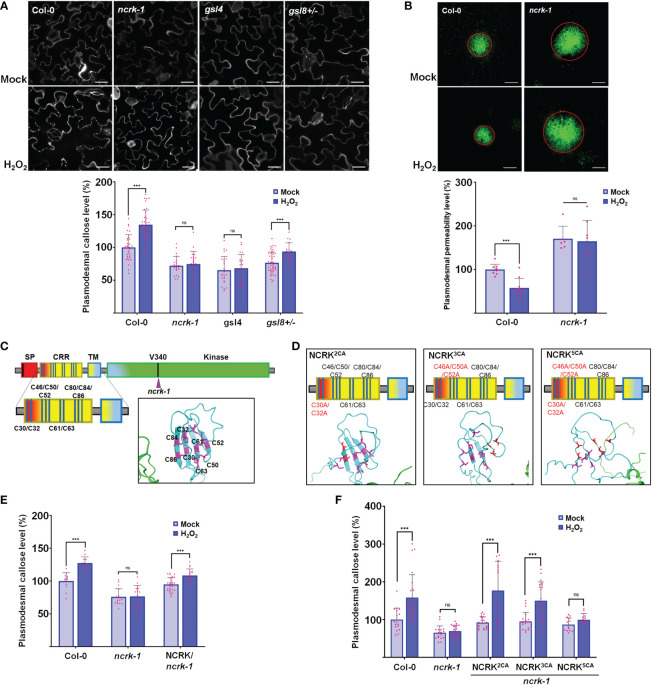
NCRK is required for H_2_O_2_-induced callose deposition. **(A)** Images of basal and ROS-dependent callose deposition in Col-0 and *ncrk-1, gsl4* and *gsl8+/-* mutant treated with H_2_O_2_ and their quantification. Scale bar: 20 μm. (n≥12). **(B)** Images of basal and ROS-dependent PD permeability of Col-0 and *ncrk-1* mutant treated with H_2_O_2_ and their quantification. Scale bar: 200 μm, (n≥12). **(C, D)** Diagram of NCRK extracellular domain and its mutants. The Cys residues are marked as magenta, the Ala residues are marked as red. The disulfide bonds of the Cys-rich motif are shown. Quantification of basal and ROS-dependent callose deposition of *ncrk-1* lines complemented with NCRK and NCRK mutants for Cys residues overexpressed using native *NCRK* promoter summarized in panel **(E, F)**, respectively. Scale bar: 20 μm. (n≥12). Data were analyzed by Student’s *t*-test. ns, not significant. ^***^ *P* < 0.001.

**Figure 3:**
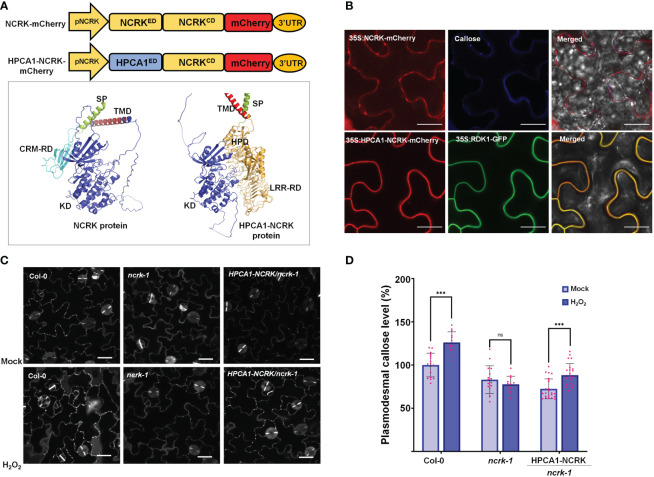
The extracellular domain of NCRK is required for basal and ROS-dependent callose deposition. **(A)** Diagram of NCRK and HPCA1/NCRK fusion protein and 3D protein structure of NCRK protein and HPCA1/NCRK fusion protein. SP, signal peptide; TMD, transmembrane domain; CRM-RD, Cys-rich motif-receptor domain; KD, kinase domain; HPD, hydrogen peroxide domain; LRR-RD, Leu-rich repeat-receptor domain. **(B)** Localization of NCRK and HPCA1-NCRK fusion protein in *N. benthamiana*. **(C)** Images of PD callose deposition of Col-0, *ncrk-1*, and HPCA1-NCRK complementation line treated with H_2_O_2_ and quantification of callose levels depicted in **(D)** (n≥12). Scale bar: 20 μm. Data were analyzed by Student’s *t*-test. ns, not significant. ^***^ *P* < 0.001.

It has been shown that the Cys residue in the ED of RLK could perceive the eH_2_O_2_ and respond to the ROS stress (Hunter et al., 2019; Wu et al., 2020). Therefore, we investigated whether NCRK could be involved in ROS-dependent callose accumulation. As described earlier, the spraying of 10 mM H_2_O_2_ on the leaf surface of wild-type plants was reported to stimulate ROS-mediated callose accumulation after 2 hours of treatment (Cui and Lee, 2016). Therefore, we focused on the role of NCRK in ROS-induced callose accumulation by eH_2_O_2_ application. We first observed that callose deposition and PD permeability of the *ncrk-1* mutant plant was impaired in 10 mM H_2_O_2_ treatment (**Figures 2A, B**). To confirm that the basal callose and ROS-induced callose impaired phenotype of *ncrk-1* was indeed caused by the NCRK mutation, we showed that NCRK could rescue the *ncrk-1* phenotype and callose accumulation was restored in the complementation lines upon eH_2_O_2_ treatment (**Figures 2E**, **3A**). NCRK contains a highly conserved and unique Cys-rich repeat sequence in the ED (**Figure 1B**). To explore whether these extracellular Cys residues are essential for eH_2_O_2_ sensing, we performed *ncrk-1* complementation using NCRK mutant, which included Cys mutations (**Figures 2C, D**). NCRK mutants with two (Cys30 and Cys32 to Ala; 2CA), three (Cys46, Cys50, and Cys52 to Ala; 3CA), or all five Cys residues to Ala (5CA) could complement the basal callose level of *ncrk-1* but not ROS-dependent callose phenotype (**Figure 2F**), suggesting that NCRK-ED might not affect basal callose accumulation, but the Cys-rich motif seems specifically involved in ROS-induced callose regulation. The HPCA1 contains the Cys residues, which could sense the eH_2_O_2_ in Arabidopsis (Wu et al., 2020). To investigate whether the Cys residues of ED in NCRK could respond to eH_2_O_2_, we pursued a domain-swapping approach replacing the ED and transmembrane domain (TMD) of NCRK with the ED and TMD of HPCA1 that yielded the chimeric H/N (HPCA1/NCRK) protein. The predicted structure of the H/N receptor was comparable with the native NCRK protein except for the ED region (**Figure 3A**). However, the chimeric H/N protein was localized at PM but not in the PD, as observed in the transient *N. benthamiana* assay (**Figure 3B**).

Further, Arabidopsis transgenic plants in the *ncrk-1* mutant background with a chimeric H/N protein driven by the endogenous NCRK promoter were generated, and eH_2_O_2_ treatment was applied (**Figure 3A**). We found that the chimeric H/N protein could not complement the callose in normal growth conditions but partially rescued the impaired callose deposition phenotype in the case of eH_2_O_2_ treatment (**Figures 3C, D**). These data suggest that NCRK-ED may serve a similar function as the HPCA1-HP domain regarding the H_2_O_2_-induced callose response. Overall, these results demonstrate the role of NCRK in eH_2_O_2_ perception at PD, and the Cys-rich motif of NCRK-ED might be involved in eH_2_O_2_-induced callose deposition.

### 2.3 NCRK requires a CML41-GSL4 complex for ROS-induced callose regulation

**Figure 4:**
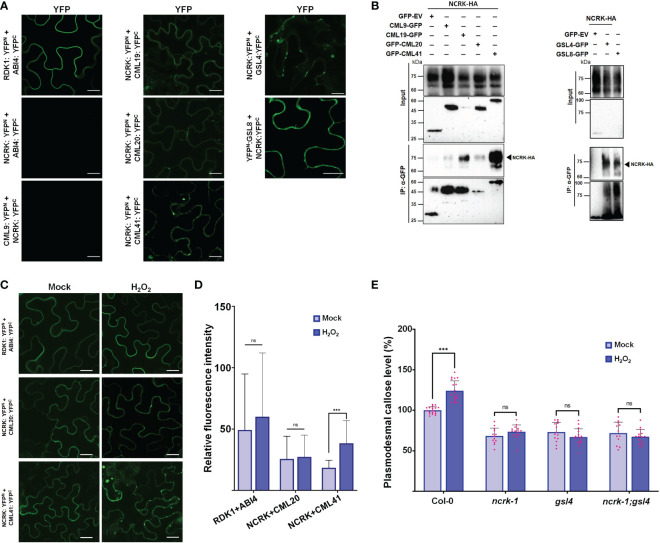
NCRK interacts with CML and GSL members. **(A)** Interaction of NCRK with its partners *in vivo* by BiFC assay. Scale bar: 20 μm. **(B)** Interaction of NCRK with its partners by Co-IP. **(C, D)** ROS-induced NCRK interaction investigated using BiFC assay and visualized by confocal microscopy. **(E)** Callose deposition of Col-0, *ncrk-1*, *gsl4* single mutant, and *ncrk gsl4* double mutant. Data were analyzed by Student’s *t*-test. ns, not significant. ^***^ *P* < 0.001.

Changes in eH_2_O_2_ and intracellular H_2_O_2_ were found to be related to an increase in Ca^2+^ and consequently activate several CML protein family members (Magnan et al., 2008; Leba et al., 2012; Wu et al., 2017; Xu et al., 2017). Also, the CML members are localized at the nucleus, cytoplasm, and PM, and some are co-localized with NCRK (**Supplementary Figure 4**). This subcellular co-localization might suggest the interaction of NCRK and the other partners. We hypothesized that NCRK might control ROS-induced callose deposition by directly interacting with CMLs members or GSL members and regulating its activity. To understand how NCRK controls the callose deposition during ROS stress and to identify the interaction partners of NCRK, we performed bimolecular fluorescent complementation (BiFC), co-immunoprecipitation (Co-IP) assay, and genetic experiments. As a positive control, RDK1-YFP^n^ and ABI4-YFP^c^ resulted in strong reconstituted YFP signals at PM (Kumar et al., 2017). However, a fluorescence signal was not observed in the leaves co-expressing NCRK-YFP^n^ and ABI4-YFP^c^ (**Figure 4A**). We detected a YFP signal on the PM when the NCRK-YFP^n^ was co-expressed with the YFP^c^ fused to CML19, CML20, CML41, GSL4, and GSL8. This data indicates that NCRK showed strong or weak interactions with several CML (CML19, CML20, CML41) and GSL (GSL4, GSL8) family members. We performed Co-IP assays to confirm the in planta interaction of NCRK with the CML and GSL family members. The result showed that NCRK coimmunoprecipitates with CML41, GSL4, and GSL8 but not with negative control (GFP) (**Figure 4B**). Consequently, BiFC and Co-IP observations revealed that NCRK strongly interacts with CML41, GSL4, and GSL8 *in vivo*. To test if the ROS can induce the interaction of NCRK and CML41, we performed the BiFC assay under the eH_2_O_2_ treatment. After 2 hours of post-spraying, the reconstituted YFP fluorescence resulting from NCRK-YFP^n^ and CML41-YFP^c^ was detected in the membrane and punctate spots. When treated with eH_2_O_2,_ the accumulation of NCRK-CML41 interaction signal intensity was increased in leaves but not in RDK1-ABI4 (positive control) and NCRK-CML20 signal intensity (**Figure 4C**). These results suggest that eH_2_O_2_ may activate NCRK, stimulating the interaction between NCRK and CML41.

Since we observed the direct interaction of NCRK and GSL members, GSL4 and GSL8, we analyzed the role of GSL members in the ROS-dependent pathway of NCRK. To determine whether GSL4 is working in the NCRK-related pathway, we generated the *ncrk gsl4* double mutant plant by crossing the *ncrk-1* and *gsl4* mutant. The callose intensity of the double mutant was similar to the *ncrk-1* and *gsl4* single mutants in normal growth and ROS-treated conditions (**Figure 4D**). It suggests that GSL4 acts in the NCRK-dependent-ROS pathway. Our data demonstrated that NCRK, CML41, and GSL4 are responsible for ROS-induced callose deposition pathways.

### 2.4 NCRK phosphorylation is required for ROS-induced callose accumulation

**Figure 5:**
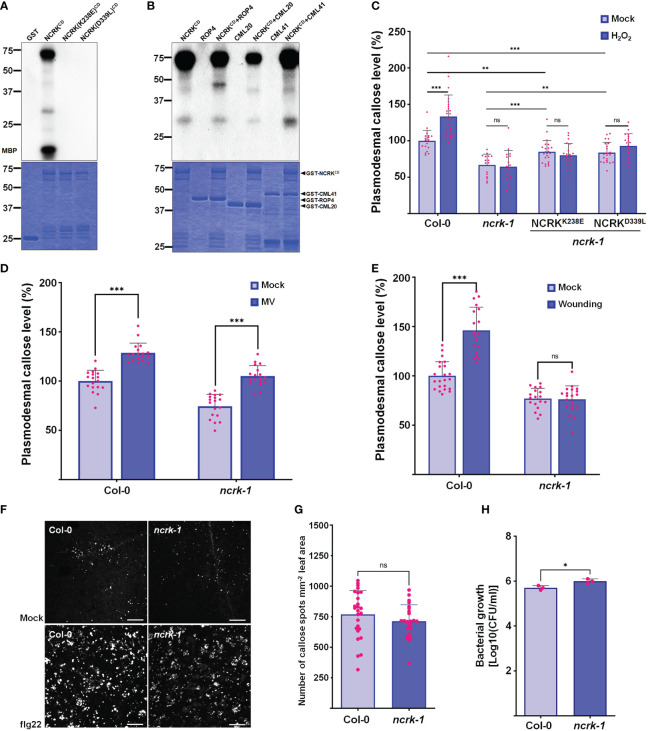
NCRK kinase activity is required for ROS-induced callose accumulation. **(A)** The kinase activity of NCRK^CD^ wild-type, NCRK(K238E)^CD^ and NCRK(D339L)^CD^ mutant proteins was analyzed by autoradiography. **(B)** NCRK phosphorylates ROP4 and CML41. **(C)** Partial complementation of the *ncrk-1* phenotype by expressing kinase-inactive variants NCRK. **(D)** Fluorescence intensity quantification of callose deposition of Col-0 and *ncrk-1* mutant following paraquat (MV) treatment. **(E)** Fluorescence intensity quantification of callose deposition in Col-0 and *ncrk-1* mutant following wounding treatment. **(F)** Callose deposition of Col-0 and *ncrk-1* mutant following flg22 treatment and **(G)** their intensity quantification. Scale bar: 200 μm. (n≥12). **(H)** Evaluation of *ncrk-1* mutant plant susceptibility to Pst DC3000 cor^-^; quantification of bacterial growth in Col-0 and *ncrk-1* upon 0 and 3 d post‐inoculation of Pst DC3000 cor^-^ suspension. Data were analyzed by Student’s *t*-test. ns, not significant. *P < 0.05, ^**^ *P* < 0.01, ^***^ *P* < 0.001.

NCRK shares similar conserved catalytic subdomains with CRK2 and HPCA1, which require kinase activity for their functions (Stone and Walker, 1995; Kornev et al., 2006) (**Supplementary Figure 5**). To explore whether NCRK (NCRK^CD^) cytoplasmic domain has kinase activity or functions as a scaffold, we prepared recombinant proteins purified from *E. coli*. We could detect the autophosphorylation and phosphorylation of myelin basic protein (MBP) only with wild-type NCRK^CD^ but not with the kinase-inactive mutants NCRK(K238E)^CD^ (ATP binding) and NCRK(D339L)^CD^ (catalytic) (**Figure 5A**). Since NCRK physically interacts with CML41, we tested whether NCRK phosphorylates the CML41 *in vitro*. The ROP4 was used as a positive control, and NCRK could phosphorylate ROP4 and CML41. NCRK also could phosphorylate CML20, but it reduces the activity of NCRK (**Figure 5B**). The *ncrk-1* transgenic plants with constructs of these two kinase inactive variants driven by the endogenous NCRK promoter were generated. The complementation lines showed partially rescued basal callose levels in mock condition but significantly reduced callose levels compared to wild-type plants under the ROS stress condition (**Figure 5C**). These data revealed that the NCRK phosphorylates CML20 and CML41 *in vitro*, and NCRK phosphorylation status influences ROS-induced callose accumulation at PD.

### 2.5 NCRK is required for wounding-induced callose deposition

Since the *ncrk-1* mutant showed the deficiency in the callose deposition under the eH_2_O_2_ treatment, we tested whether ROS production triggered by another abiotic and biotic stress in the plant could affect the callose deposition in NCRK mutant background. Previously, herbicide methyl viologen (MV) treatment was shown to induce ROS production in chloroplast and mitochondria (Cui et al., 2019). The chloroplast and peroxisomes are the primary sources of intracellular ROS in plants (Noctor et al., 2002). To determine the effect of MV application on callose deposition, wild-type plants were air sprayed with different MV concentrations (0.1, 1, and 5 μM). Wild-type plants sprayed with 5 μM of MV showed considerably higher callose deposition at 2 hours post-treatment (**Supplementary Figure 6**). Similarly, a higher level of callose accumulation was observed in the *ncrk-1* mutant plants sprayed with 5 μM of MV (**Figure 5D**). This data revealed that NCRK is independent of MV-induced callose deposition. Next, we tested whether NCRK is required for apoplastic ROS-induced callose deposition. Since GSL4 is specifically responsible for apoplasmic ROS-mediated PD regulation during wounding stress and NCRK acts as a mediator of GSL4 in eH_2_O_2_ response, we quantified callose level after mechanical wounding to wild-type and *ncrk-1* mutant plants. This experiment showed the *ncrk-1* mutant is impaired to gain of callose intensity within 2 hours post-wounding (**Figure 5E**). These data suggest that NCRK plays a role in wounding-dependent callose deposition.

The previous study has shown that CML41 is a responsive component of callose deposition-pathogen defense, and an flg22 (*P. syringae*) also triggers apoplastic ROS in plant immunity (Xu et al., 2017). To examine the function of NCRK mediating ROS-induced plant signaling network during pathogenic stress, we infiltrated wild-type and *ncrk-1* mutant plants with flg22 to assess the impact of the altered callose regulation. After 24 hours post-infiltration, PD callose of *ncrk-1* mutant plants was slightly reduced compared to wild-type plants (**Figures 5F, G**). Additionally, we focused on assessing the significance of NCRK activity in bacterial resistance by surface inoculation with *P. syringae*. After three days post-infection (3 dpi), *ncrk-1* mutant plants showed increased bacterial growth compared to wild-type plants, indicating that NCRK might be involved in an flg22-induced PD closure pathway.

## 3 Discussion

In this study, we have shown that NCRK accumulates at the plasmodesmal region and regulates the callose deposition and, thereby, PD permeability. NCRK also contributes to maintaining ROS-induced callose deposition upon eH_2_O_2_ stress. The expression of NCRK mutated at five Cys residues with Ala could not complement *ncrk-1* callose level after ROS treatment, suggesting Cys residues of NCRK-ED are essential for ROS-dependent callose accumulation. As the H/N chimeric protein could rescue the *ncrk-1* phenotype, the NCRK-ED might serve as an eH_2_O_2_ receptor. Furthermore, NCRK has kinase activity, which is required for ROS-induced callose deposition. We also demonstrated that NCRK could physically interact with GSL4 and CML41 and mediate ROS-dependent callose response.

NCRK is the plasmodesmal RLK responsible for basal callose deposition, as demonstrated in the callose staining assay and Drop-ANd-See (DANS) assay (**Figures 1D, E**). Previous reports have shown that basal PD permeability was controlled by GSL4 and GSL8 in different callose-related pathways (Cui and Lee, 2016). The plant phenotype and callose level of the *ncrk-1* mutant were like *gsl4* mutants (**Figures 1F**, **2A**), indicating that NCRK and GSL4 have similar functions with respect to callose regulation. GSL4 contributes to the maintenance of basal PD permeability, which relies on PDLP5 function (Cui and Lee, 2016), whereas GSL8 physically interacts with PDLP5 (Saatian et al., 2018) and controls basal PD callose but does not need it for functioning in ROS-dependent PD permeability regulation (Cui and Lee, 2016). Therefore, GSL4 and GSL8 may interact directly or indirectly with other proteins to control basal PD permeability (Iswanto et al., 2022). Here, we found that the NCRK interacts with GSL4 and GSL8 (**Figure 4B**), suggesting basal PD permeability could be mediated by NCRK interactions with GSL4 or GSL8 and may be linked with PDLP5 functioning. Thus, NCRK could interact with PDLP5 to regulate callose synthase activity, an exciting aspect to explore in future work.

Previously, the yeast two-hybrid assay showed NCRK interaction with ROP11 (Molendijk et al., 2008). ROP11 also interacts with RBOHF and regulates ROS production (Yan et al., 2016), indicating the possible involvement of NCRK in ROS-induced callose response. To test this hypothesis, we performed a callose staining assay together with eH_2_O_2_ spraying to examine the callose level in *ncrk-1* mutant leaves. Using this assay, we compared the plasmodesmal callose of *ncrk-1* with wild-type, *gsl4*, and *gsl8* heterozygous mutants. The callose deposition of *ncrk-1* mutant plants was impaired in eH_2_O_2_ response (**Figure 2A**). Moreover, the *ncrk gls4* double mutant also showed an impaired callose deposition pattern similar to the *ncrk-1 or gls4* single mutant (**Figure 4E**). In summary, NCRK and GSL4 function in the basal and ROS-dependent callose deposition pathways.

As discussed earlier, Cys-rich motifs have been proposed as a candidate for ROS sensors responsible for most of the communication between the apoplast and intracellular environment in various stress responses (Hunter et al., 2019; Kimura et al., 2020; Wu et al., 2020). It is also suggested that the activity of a candidate H_2_O_2_ receptor is based on their extracellular Cys-rich motifs located at PD to respond rapidly and trigger the callose defense upon ROS stress (Cheval et al., 2020; Castro et al., 2021). Our callose staining assay showed that the 5CA (but not 3CA or 2CA) mutant failed to retain the function of callose accumulation in ROS response (**Figure 2F**). It is reported that the conserved Cys motif of Cys-rich receptor-like kinases could form the disulfide bridges and control the thiol redox regulation (Wrzaczek et al., 2010; Idänheimo et al., 2014). The predicted NCRK protein structure revealed the orientation of Cys residues of NCRK-ED could form disulfide bonds (**Figure 2C**). These disulfide bonds could not be formed when the five Cys residues were mutated with Ala (**Figure 2D**), indicating that Cys residues might be sites of eH_2_O_2_ sensing.

Furthermore, H/N chimeric complementation plant can respond to eH_2_O_2_ and trigger callose deposition in the *ncrk-1* mutant background (**Figure 3D**). Altogether, our data suggests that the Cys-rich motif of NCRK serves as an ROS sensor, likewise the HP domain of HPCA1 but needs more investigations. Therefore, considering the disulfide bonds are required for ROS-induced callose deposition, and NCRK-ED function is similar to that of HPCA1-ED, it is tempting to speculate that Cys residues of NCRK-ED might also be modified by eH_2_O_2_. Thus, further characterization of reduced-oxidized states of the Cys-rich motif will provide molecular insights into NCRK functioning in the ROS sensing mechanism.

Previous studies showed that the CRK2 could translocate to PD, phosphorylate GSL6 (Cals1), and subsequently promote callose deposition (Hunter et al., 2019). No significant changes were observed in callose accumulation between non-treated and NaCl-treated CRK2 kinase-dead mutant lines. Therefore, CRK2 kinase activity is essential for salt-induced callose response. Similarly, our results about quantification of callose deposition upon eH_2_O_2_ stress or mock conditions of kinase-inactive lines showed no significant differences (**Figure 5B**). In addition, the basal callose level of kinase-inactive plants was not fully recovered compared to wild-type under normal growth conditions. These results suggest that NCRK kinase activity is crucial in basal and ROS-dependent callose regulation. Notably, CRK2 directly interacts with the GSL6 and phosphorylates it (Hunter et al., 2019). Hence, considering the interaction of NCRK with GSL4 and GSL8, it will be interesting to test whether NCRK might phosphorylate GSL4 or GSL8. Moreover, NCRK shares the conserved Threonine (Thr) residues similar to the kinase domains of CRK2 and HPCA1 that were previously confirmed as primary phosphorylation sites (**Supplement Figure 5**). Thus, the role of Thr residues of NCRK could be studied to know their functions in phosphorylation-mediated regulation of GSL4 or GSL8 during the H_2_O_2_ stress.

CML41 is a PD-localized Ca^2+^-binding protein required for plant defense response during pathogen attack (Xu et al., 2017). In addition, the interaction strength between NCRK and CML41 intensified following ROS treatment (**Figures 4C, D**). Since CML41 (**Figures 4C, D**) and GSL4 (**Figure 4E**) mediates ROS-induced callose accumulation, which is dependent on NCRK function, the combination of NCRK and CML41 might incorporate with GSL4 to modulate the callose accumulation in response to ROS stress. We found herbicide (MV)-induced callose deposition was independent of NCRK functioning. On the other hand, biotic stressor (flg22) treatment showed slightly reduced callose deposition in NCRK mutant background compared with wild-type Col-0 (**Figures 5D, F–H**). We also demonstrated that GSL4 requires NCRK to respond to the mechanical injury (wounding)-induced stress and subsequently callose-mediated PD regulation (**Figure 5E**). Overall, comparative analysis of wild-type and *ncrk* mutant plants indicated that NCRK may or may not be directly involved in the stress response pathway, depending on the stress type. Given that the Arabidopsis genome encodes 12 GSL and 50 CML family members, NCRK interaction with a combination of these different GSL or CML family members may modify the protein conformation and transduce cellular signals generated by various stresses to mediate plant defense responses.

## 4 Materials and methods

### 4.1 Plant material and growth condition

Seedlings of *Arabidopsis thaliana*, ecotype Columbia-0 (Col-0) were grown in soil or in petri dishes in half-strength Murashige and Skoog (MS) salts (Duchefa Biochemie, P.O. Box 809 2003 RV Haarlem, The Netherlands), 1.0% (w/v) sucrose (BioShop, Canada Inc., 5480 Mainway, Burlington, Ontario L7L 6A4, Canada), and 0.6% (w/v) agar (Duchefa Biochemie) in controlled environmental rooms or plant growth chambers (20-22°C). The fluency rate of white light was approximately 100 μmol m^-2^s^-1^. The photoperiods were 16 hours/8 hours light/dark cycles. For seedling culture, seeds were sterilized with 20% bleach for 5 min, washed with sterilized water, and then sown on soil or half-strength MS media, placed at 4°C for 3 days in the dark, and then transferred to growth rooms or chambers. The hydroponic assay was performed in half-strength of MS media, and plants were grown at the culture room condition (22°C under 16 hours/8 hours light/dark cycles). T-DNA insertion mutants were obtained from the Arabidopsis Biological Resource Center (https://abrc.osu.edu). The T-DNA insertion mutants of NCRK (SALK_045757 and SALK_202953 designated as *ncrk-1* and *ncrk-2*, respectively) identified by T-DNA insertion-based PCR using T-DNA left-border primer (LBb1.3) and two pairs of NCRK-specific primers (LP1-RP1 and LP2-RP2), respectively.

### 4.2 Constructs and transgenic lines

Golden Gate cloning system (Werner et al., 2012) was used to generate *pNCRK::NCRK-mCherry*, *pNCRK::NCRK(C30A/C32A)-mCherry*, *pNCRK::NCRK(C46A/C50A/C52A)-mCherry*, *pNCRK::NCRK(C30A/C32A/C46A/C50A/C52A)-mCherry*, *pNCRK::NCRK^K238E^-mCherry, pNCRK::NCRK^D339L^-mCherry, pNCRK::HPCA1^ED^-NCRK^CD^-mCherry*. The full-length NCRK genomic region (2 kb upstream and 1 kb downstream of the NCRK coding region), HPCA1 ED region (2 kb), NCRK promoter region (2 kb), and NCRK1 full-length genomic were amplified by PCR from genomic DNA. These fragments were cloned into the pAGM4723 expression vector (Addgene #48015). Transgenic Arabidopsis lines were generated by *Agrobacterium*-mediated transformation (Zhang et al., 2006), and homozygous transgenic T3 lines carrying a single insertion were used. The *ncrk-1* mutant was complemented by a genomic fragment and NCRK-mCherry driven by the native NCRK promoter. The site-directed mutagenesis was used to introduce Cys-to-Ala mutations into the *pNCRK::NCRK-mCherry* plasmid. For the *pNCRK::GFP-GUS*, *p35S::CML19-GFP*, *p35S::CML19-GFP*, *p35S::CML20-GFP*, was cloned by Gateway cloning. The NCRK promoter fragment, CML9, CML19, CML20, and CML41 cDNA were amplified from genomic DNA. These fragments were moved into pDONR207 plasmid (Invitrogen, 5791 Van Allen Way PO Box 6482 Carlsbad, California 92008, USA) and then cloned into the Gateway binary vector pKGWFS7, pMDC43, and pMDC83, respectively.

### 4.3 Phylogenetic analysis and protein structure analysis

A phylogenetic tree was created by multiple sequence alignment method using MEGA (v11.0.9) (Tamura et al., 2021), in which bootstrap was performed with 1,000 replicates, and gaps were treated as pairwise deletions. The neighbor-joining method performed a bootstrap with 1,000 replicates for the phylogenetic trees. Multiple sequence alignments of the full-length amino acid sequence of **Figure 1B** (*OsNCRK*, *GRMZM2G063897*, *Glyma.11G144800*, *Glyma.12G077300*, and *AtNCRK*) and **Supplementary Figure S5** (CRK2, HPCA, and NCRK) were aligned by ClustalW (https://www.ebi.ac.uk/Tools/msa/clustalo) (Sievers et al., 2011). The protein structure of NCRK, H/N fusion protein, and NCRK-ED were predicted by AlphaFold2 using MMseq2 (Mirdita et al., 2022). The 3D structure was visualized by the PyMOL program.

### 4.4 Histochemical GUS activity analysis

Histochemical GUS staining of the NCRK promoter-driven GUS (*pNCRK::GFP-GUS*) transgenic lines was detected by infiltration with 5-bromo-4-chloro-3indolyl-b-D-glucuronic acid (X-Gluc), as described in an earlier report (Kumar et al., 2017). Briefly, plant materials were incubated at 37°C overnight in a GUS staining buffer containing 2 mM X-Gluc, 2mM potassium ferricyanide, 2 mM potassium ferrocyanide, 0.2% Triton X-100, and 50 mM potassium phosphate buffer, pH 7.0 and followed by chlorophyll clearing in 75% ethanol. GUS images were taken using a stereomicroscope.

### 4.5 NCRK-mCherry subcellular localization analysis and BiFC assays

For analysis of NCRK-mCherry, HPCA1/NCRK-mCherry in stable transgenic Arabidopsis lines, seedlings grown in half-strength MS media in Petri dishes for 7 days were subjected to mCherry confocal imaging with the FV1000 or FV1000MPE confocal microscope (Olympus, Japan). Data represent more than 3 independent lines examined, which displayed similar mCherry subcellular localization. To analyze wild-type NCRK and HPCA1/NCRK protein subcellular location, the transient expression of fluorescent fusion proteins in *N. benthamiana* leaf tissue was performed as previously described (Iswanto et al., 2020). The full-length coding sequences of NCRK, HPCA1/NCRK were amplified from Col-0 and fused with p35S and mCherry into pAGM4723 vector by Golden Gate cloning. For BiFC assays, LR recombination of appropriated coding sequence regions (NCRK, CML9, CML19, CML20, CML41, GSL4, and GSL8) in pDONR207 were performed with the split-YFP destination vectors pDEST-^GW^VYNE/pDEST-VYNE^®GW^, which allow C-terminal fusions with C-terminal YFP moieties (Gehl et al., 2009). *Agrobacterium tumefaciens* strain GV3101 containing the binary plasmid was grown in LB medium with the appropriate antibiotics at 28°C for 24 hours for the primary culture and then subculture at 28°C for 12 hours. The cells were centrifuged for 15 min at 3500 rpm, resuspended to a final OD_600_ of 0.8, and infiltrated into tobacco leaves by a needle-less syringe. Leaf pieces were viewed under a confocal microscope after 2-3 days.

### 4.6 Protein extraction and co-immunoprecipitation

For Co-IP in *N. benthamiana*, Golden Gate was used to clone PDRLK1 full-length genomic to generate HA-tagged fusions. pDONR207-*CML19, CML20, CML41* and *CML9, GSL8* and *ROP4* were recombined into pMDC43 and pMCD83, respectively, to generate GFP-tagged fusions by Gateway cloning. To clone the *p35S::GSL4-GFP* construct, GFP with *Nos* terminator was amplified with additionally flanked with restriction sites for SmaI and SpeI. The amplicon was excised and cloned into the construct pDEST-^GW^VYNE-GSL4 *via* SmaI/SpeI. These constructs were electroporated into *A. tumefaciens* strain GV3101 (pMP90). *Agrobacterium* suspensions carrying *p35S::PDRLK1-HA*, *p35S::GFP-CML19*, *p35S::GFP-CML20*, and *p35S::GFP-CML41, p35S::GSL8-GFP, pDEST::GSL4-GFP* were infiltrated in various combinations into *N. benthamiana* leaves (OD_600_ = 0.3). One gram of leaf tissue was harvested at 48-72 hpi, and total protein was resuspended in 1 ml IP buffer (100 mM Tris-HCl pH 7.5, 150 mM NaCl, 1 mM EDTA, 10% glycerol, 3 mM DTT, 1 mM Na_2_MoO_4_, 1.5 mM sodium orthovanadate [Na_3_VO_4_], 2 mM sodium fluoride [NaF], 1 mM phenylmethylsulfonyl fluoride [PMSF], 50 µM MG132, a complete protease inhibitor [Roche] and 0.5% IGEPAL). The samples were clarified by centrifugation at 13,000 rpm, at 4°C for 10 min twice. The supernatant was incubated with 10 μl of protein A agarose incubated with anti-GFP antibody for 2 hours at 4°C with 360° rotating shaker. Beads were washed five times with IP buffer. 40 μl of 2× SDS sample buffer was added to the beads, and the beads were heated at 70°C for 15 min and subjected to further SDS-PAGE and immunoblotting analysis (Kadota et al., 2016; Kumar et al., 2017).

### 4.7 DANS assays, plasmodesmal callose quantification

For hypocotyl experiments, five-day-old, etiolated Arabidopsis seedlings were first cut at the base of the hook using a razor blade (Iswanto et al., 2020). A coverslip was placed between each hypocotyl cut surface and the MS agar plate. For dye loading, individual agar blocks containing 8-hydroxypyrene-1,3,6-trisulfonic acid trisodium salt (5 mg/ml) were placed on the cut hypocotyl surface. After a 5 min loading period, seedlings were washed in water for 15 min, and then fluorescent probe movements were evaluated by confocal microscopy. For leave experiments, intact 2.5 to 3.5-week-old Arabidopsis plants were air sprayed once with H_2_O_2_, MV, or wounding. Following a designated incubation time, the fifth and sixth entire leaves were subjected to DANS assays. DANS dye-loading assay using 1 mM 5(6)-carboxy-2’-7’-dichlorofluorescein diacetate for cell-to-cell movement assay as described (Cui and Lee, 2016). Briefly, the dye was loaded as a 1 µl droplet on the central regions of the adaxial side of each half-leaf blade, followed by confocal imaging of the abaxial side after 5 min loading and rinsing with a distilled water. Plasmodesmal callose staining was performed by staining the mature leaves or hypocotyl, for 1-1.5 hours in the dark before confocal imaging, with 0.02% aniline blue solution containing 0.6 M glycine-NaOH, pH 9.5, and 0.001% silwet L-77 (Han et al., 2014; Cui and Lee, 2016). Quantifying aniline blue stained plasmodesmal callose levels was performed using ImageJ as described before (Zavaliev and Epel, 2015) by automatically extracting plasmodesmata-associated fluorescent spots along the cell boundaries of epidermal leaf pavement cells and plotting fluorescence intensity per unit area.

### 4.8 Chemical treatments, mechanical wounding, and bacterial infection

All chemical treatments were performed by spraying them on intact and mature plants at various concentrations before DANS assays or plasmodesmal callose quantification. Mechanical wounding was done by snipping the tip of the fifth or sixth leaf with a pair of sharp forceps (Cui and Lee, 2016). Infected plants were grown in the plant chamber and subjected to DANS assays or plasmodesmal callose quantification 48 hours after infection. For flg22 treatment, either distilled water or distilled water containing 1 µM of flg22 was infiltrated into rosette leaves. After 24 hours, the infiltrated leaves were incubated in callose staining buffer (aniline blue) for 1-1.5 hours in the dark (Xu et al., 2017). For bacterial growth curve assay, we performed infections by surface inoculation with the less virulent, coronatine-deficient strain DC3000 (cor^-^). Briefly, Pst cultured at 28°C and resuspended in MgCl_2_ to final A_600_ nm between 0.02 and 0.04 were generously infiltrated onto leaf abaxial surfaces of 5-6-week‐old plants. Plants were covered, and leaf discs were taken 3 dpi from three leaves per plant, with three plants per genotype per independent trial. Bacterial growth was assessed by colony counting (Kadota et al., 2014).

### 4.9 Confocal microscopy and image processing

For detection of aniline blue-stained plasmodesmal callose, confocal images were taken under a 100X UplanXApo objective with oil, using 405-nm laser excitation with a 420 to 480 nm emission filter. Free or tagged mCherry was imaged with 543 nm laser excitation and a 587 to 683 nm bandpass emission filter. GFP was imaged with 488 nm laser excitation and a 505 to 550 nm bass pass emission filter. DANS and HPTS assay images were taken under a 10X UplanXApo objective using 488 nm Argon laser excitation, with a 505 to 550 nm band pass emission filter. All confocal images were acquired and processed using Olympus FV1000MPE or FV3000 confocal microscope or ImageJ software (Iswanto et al., 2020).

### 4.10 Recombinant protein purification and kinase assay

Recombinant protein purification and kinase assays were conducted as previously described (Harper and Speicher, 2011). NCRK^CD^ (wild type, (K238E)^CD^ and (D339L)^CD^) and other substrates (CML20, CML41, and ROP4) were amplified and ligated into the pGEX4T-1 vector containing GST tag. Overexpression of those proteins in *E. coli* strain BL21(DE3) and purification on glutathione sepharose 4B R10 (GE Healthcare, USA) were conducted as described. For the kinase assay, these GST-fusion cytoplasmic domain kinase proteins (3 μg), GST (1 μg; negative control) MBP (0.5 μg; positive control) were incubated in kinase reaction buffer (25 mM Tris-HCl, pH 7.5, 1 mM DTT, 20 mM MgCl_2_, 2 mM MnCl_2_, and 50 μM [γ-^32^P] ATP) and initiated using 1 μCi [γ-^32^P] ATP. The final reaction volume was 20 μl. After 30 min at 30°C, the reaction was stopped by adding 6 μL of 4 × SDS sample buffer and boiling for 5 min. Samples containing 1 μg of protein from each autophosphorylation reaction were separated by SDS-PAGE, and the gel was stained, dried, and autoradiographed (Kim et al., 2017).

### 4.11 Statistical analysis

Column plots were created using GraphPad Prism version 9.0.0 for windows, GraphPad Software, San Diego, California, USA (www.graphpad.com). Student’s *t*-test was performed to test the statistical significance of differences.

## Data availability statement

The original contributions presented in the study are included in the article/**Supplementary Material**, further inquiries can be directed to the corresponding author/s.

## Author contributions

MHV and J-YK conceived the idea and designed the experiments. YJ and MHV designed experiments, performed experiments, analyzed data, and wrote the manuscript. TKH, SB, RK, DT, and ABBI performed the experiments. RMS contributed to the interpretation and draft of the output. RMS and J-YK revised the manuscript. All authors read and approved the final manuscript.
