# Supplementary material for: ROS-mediated plasmodesmal regulation requires a network of an Arabidopsis receptor-like kinase, calmodulin-like proteins, and callose synthases
Source: Front Plant Sci. 2023 Jan 19;13:1107224. doi: 10.3389/fpls.2022.1107224 (PMC9893415; doi:10.3389/fpls.2022.1107224)
Supplement: Supplementary file 1 [file DataSheet_1.pdf]

Supplementary Material

ROS-mediated plasmodesmal regulation requires a network of an Arabidopsis receptor-like kinase, calmodulin-like proteins, and callose synthases

Minh Huy Vu[1], Tae Kyung Hyun[2], Sungwha Bahk[1], Yeonhwa Jo[1, 3], Ritesh Kumar[1,4], Dhinesh Kumar[1,5], Arya Bagus Boedi Iswanto[1], Woo Sik Chung[1,6], Rahul Mahadev Shelake[1,*], Jae-Yean Kim[1,6,7,*]

Table of Contents

Sr. No.	Title	Page
1	Supplementary Figure S1. NCRK is a PD-localized protein.	2
2	Supplementary Figure S2. NCRK ubiquitously expressed in whole plant.	3
3	Supplementary Figure S3. NCRK regulates basal callose level and PD permeability.	4
4	Supplementary Figure S4. The cellular localization of free GFP, CML9-GFP, CML19-GFP, GFP-CML20, GFP-CML41, and GFP-ROP4 in *N. benthamiana* by transient assay.	5
5	Supplementary Figure S5. Highly conserved kinase activity and phosphorylated Thr residues in the catalytic core of the kinase domains of NCRK, CRK2, and HPCA1.	6
6	Supplementary Figure S6. Fluorescence intensity quantification of callose deposition of Col-0 following a 2 h paraquat (MV) treatment.	7
7	Supplement Table 1. List of primers used in this study.	8
8	Supplement Table 2. n values for experiments with unequal sample size. n is the number of images analyzed.	12

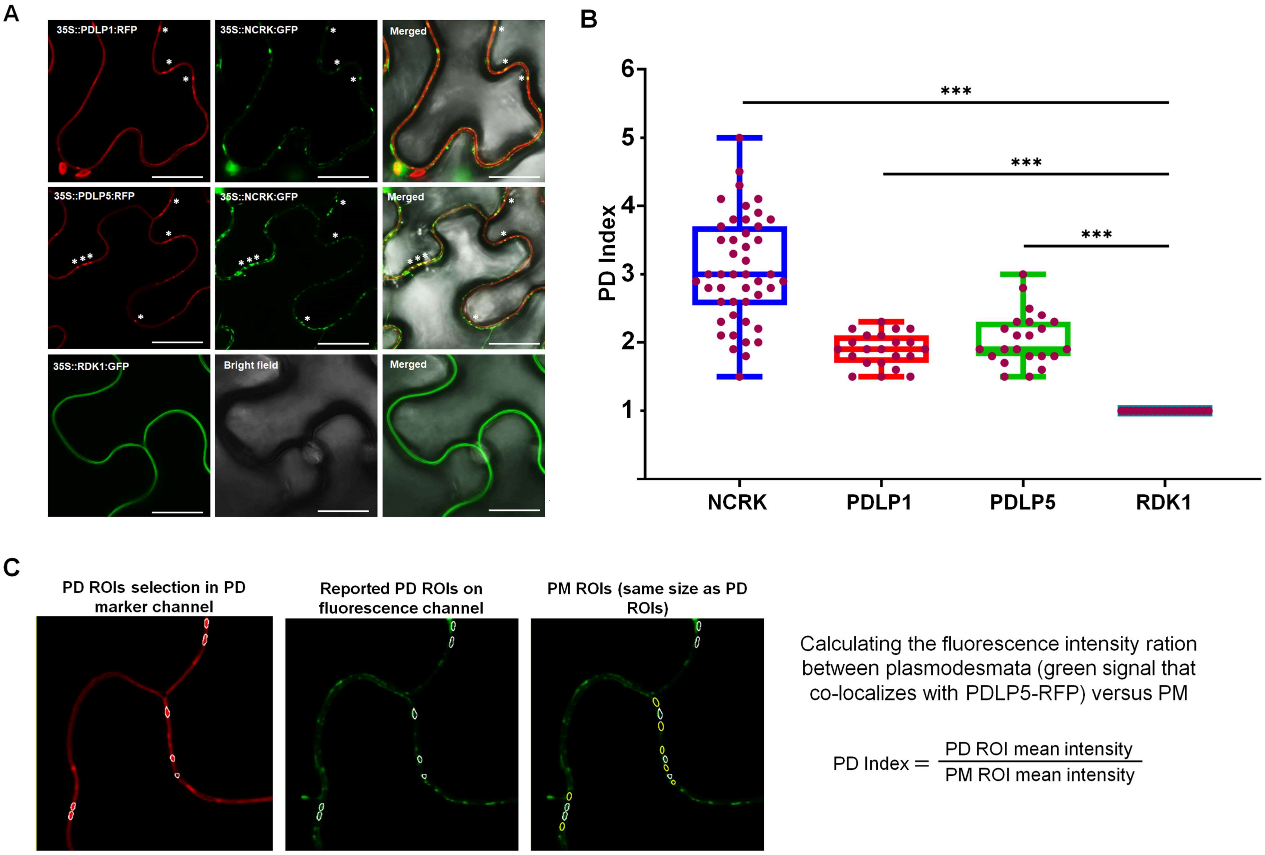

Supplementary Figure S1. NCRK is a PD-localized protein. (A) Co-localization of NCRK with PDLP1 and PDLP5 at PD spot in *N. benthamiana*. Co-localization areas are indicated by a white asterisk **(B)** Quantification of the PD index of NCRK-GFP, PDLP1-RFP, PDLP5-RFP, and RDK1-GFP in *N. benthamiana*. **(C)** Enrichment at PD versus the PM was quantified by the PD index, which corresponds to the fluorescence intensity ratio of the NCRK at PD versus the PM in control. Scale bar: 20 μm. Data was analyzed by Student's *t*-test. ns, no significant. $^{***}P < 0.001$.

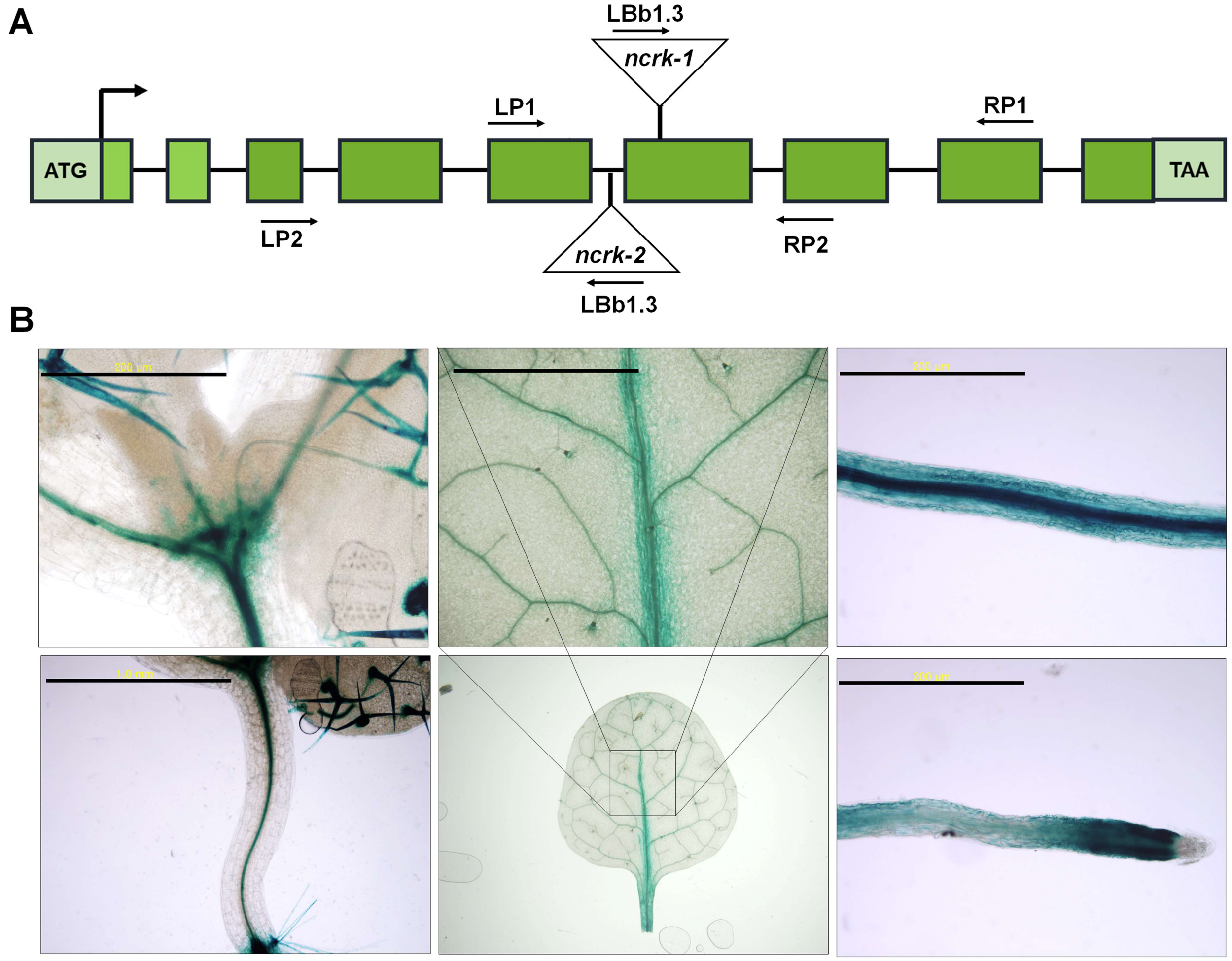

Supplementary Figure S2. NCRK ubiquitously expressed in whole plant. (A) Schematic of T-DNA NCRK insertion. T-DNA insertion is shown as a triangle (*ncrk-1*) or an inverted triangle (*ncrk-2*). LP1 and RP1 or LP2 and RP2 are the forward and reverse primer pairs, respectively, used for genotyping; LBb1.3, primer specific to the T-DNA left border. **(B)** The expression patterns of *pNCRK::GUS* in transgenic *Arabidopsis* seedlings grown on half MS medium for 5 days. Scale bar: 200 μm.

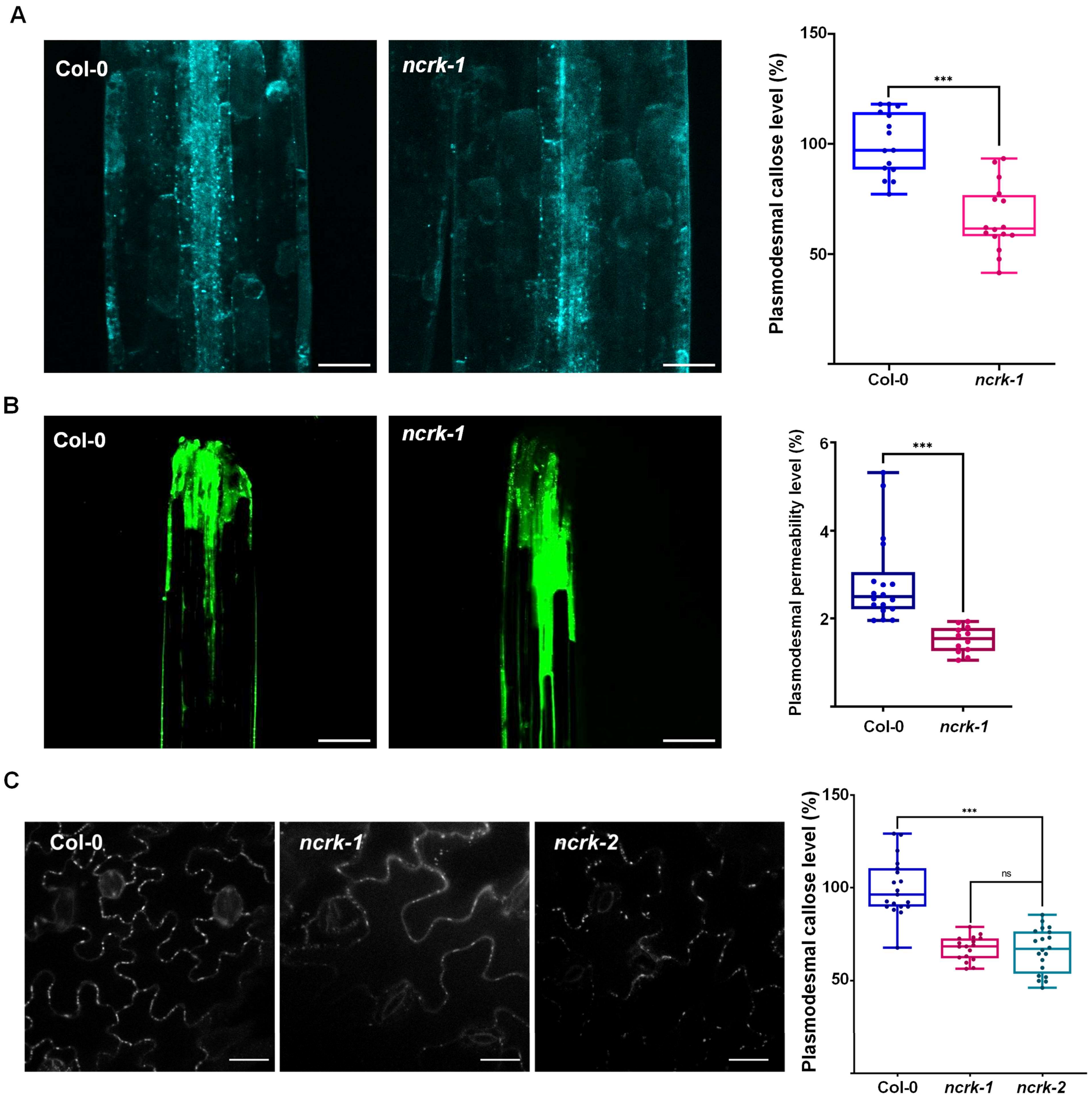

Supplementary Figure S3. NCRK regulates basal callose level and PD permeability. (A) Callose staining in the hypocotyl. PD callose was stained and measured the relative callose intensity. Scale bar: 20 µm. **(B)** PD permeability in the hypocotyl. PD permeability was traced by HPTS and measured the green fluorescence intensity. Scale bar: 200 µm. **(C)** Callose staining in the leaf tissue. PD callose was stained and measured the relative callose intensity. Scale bar: 20 µm. Data was analyzed by Student's *t*-test. ns, no significant. $^{***}P < 0.001$.

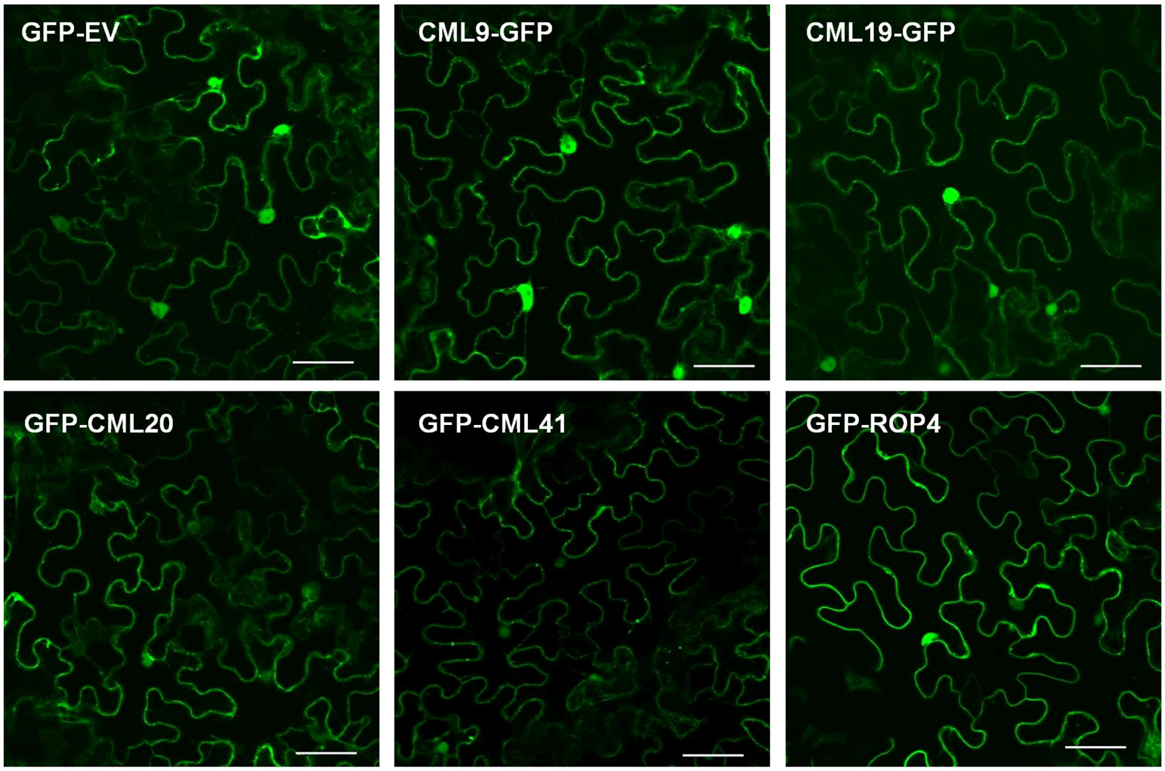

Supplementary Figure S4. The cellular localization of free GFP, CML9-GFP, CML19-GFP, GFP-CML20, GFP-CML41, and GFP-ROP4 in *N. benthamiana* by transient assay. Confocal microscopy images of epidermal *N. benthamiana* cells infiltrated with an *Agrobacterium* suspension harboring constructs empty vector (EV)-GFP, CML9-GFP, CML19-GFP, GFP-CML20, GFP-CML41 and GFL-ROP4. Scale bar: 20 µm.

```
                ATP binding
                     ↓
NCRK     KTAAIKRLNTPKGDDTDTLFSTEVELLSRLHHYHVVPLIGYCSEFHGKHAERLLVFEYMS 292
HPCA1    QLIAIKRAQQGS-LQGGLEFKTEIELLSRVHHKNVVRLLGFCFDR----NEQMLVYEYIS 708
CRK2     RDIAVKRLFFNN-RHRATDFYNEVNMISTVEHKNLVRLLGCSCSG----PESLLVYEYLQ 402
         :  *:**    .  .    * .*::::* :.* ::* *:* . .      * :**:**:.

                                                      Catalytic
                                                          ↓
NCRK     YGSLRDC-LDGELGEKMTWNIRISVALGAARGLEYLHEAAAPRILHRDVKSTNILLDENW 351
HPCA1    NGSLKDS-LSGKSGIRLDWTRRLKIALGSGKGLAYLHELADPPIIHRDIKSNNILLDENL 767
CRK2     NKSLDRFIFDVNRGKTLDWQRRYTIIVGTAEGLVYLHEQSSVKIIHRDIKASNILLDSKL 462
           **    :. : *  : *  * .: :*:..** **** :   *:***:*:.*****.:

                              Phosphorylated Thr residues
NCRK     HAKITDLGMAKCLSSDGLQSGSSSPTTGLQGTFGYFAPEYAIAGCASQMSDVFSFGVVLL 411
HPCA1    TAKVADFGLSKLVGDPE----KTHVTTQVKGTMGYLDPEYYMTNQLTEKSDVYGFGVVLL 823
CRK2     QAKIADFGLARSFQDD-----KSHISTAIAGTLGYMAPEYLAHGQLTEMVDVYSFGVLVL 517
          **::*:*::: . .      .:  :* : **:**: ***   .  ::  **:.***::*
```

Supplementary Figure S5. Highly conserved kinase activity and phosphorylated Thr residues in the catalytic core of the kinase domains of NCRK, CRK2, and HPCA1. Bold residues (blue and red) are highly conserved in active kinases. Amino acid residues (orange) are confirmed phosphorylation sites. The phosphorylation of these Thr residues is required for kinase activation in the presence of H_2O_2.

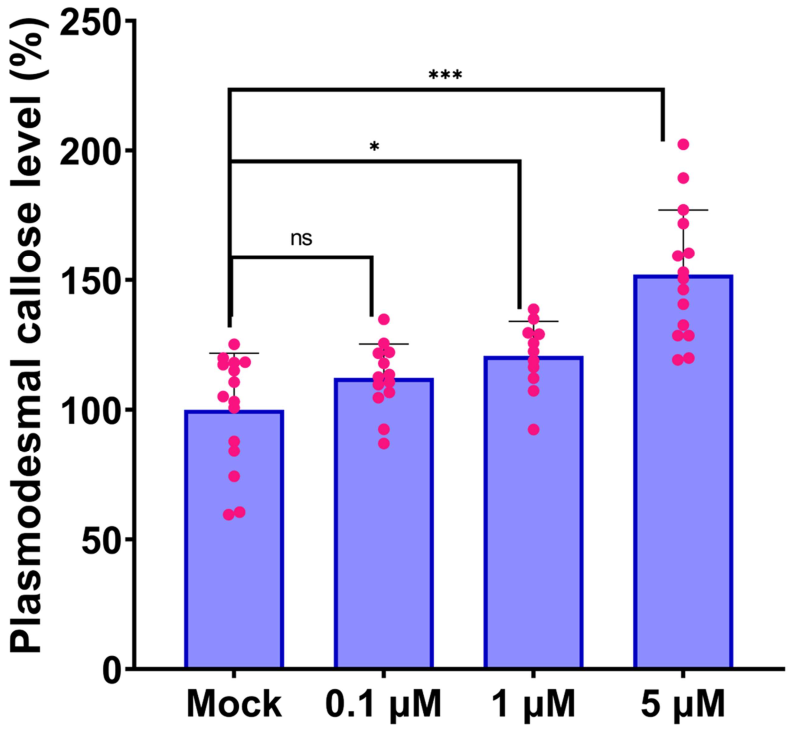

Supplementary Figure S6. Fluorescence intensity quantification of callose deposition of Col-0 following a 2 hours paraquat (MV) treatment. Callose staining assay was performed at 2 hours after single spray application of MV. Data was analyzed by Student's *t*-test. ns, no significant. $^{*}P < 0.05$, $^{***}P < 0.001$.

Supplement Table 1. List of primers used in this study

Primer name	Sequence (5'→3')	Purpose
LBb1.3	ATTTTGCCGATTTCGGAAC	Genotyping of T-DNA insertion
SALK_045757 LP (RP1)	CTGGATGATGTGTCTGGTGTG	Genotyping of *ncrk-1* allele
SALK_045757 RP (LP1)	TTTACCGTGGACAGCTCAAAG	
salk_202953 RP (RP2)	TGAACCGCTTTGTAAACCATC	Genotyping of *ncrk-2* allele
salk_202953 LP (LP2)	TCATGCTTCACTTCATGCAAC	
FP1_NCRK_2C-A	TAAGCAACTATACA GCTACGGCTTTCTC TTCGGGC	Cloning NCRK mutation by site-direct mutagenesis PCR
RP1_NCRK_2C-A	GAGAAA GCCGTAGC TGTATAGTTGCTTA CACCTAACAAAG	
FP2_NCRK_3C-A	TTCCACATCAGCTAATGCTAGACCAGGTT CGTTTT	
RP2_NCRK_3C-A	GATATCCTGGAATCTAACGCTTCCACATC A	
FP_NCRK_K238D	AAAACCGCTGCGATCGATCGTCTAAATA CCCCTAA	
RP_NCRK_K238D	AGACGATCGATCGCAGCGGTTTTACCGTC TTTGAGCT	
FP_NCRK_D339L	GAATCTTACACCGACTTGTGAAATCCACA AATATTCT	
RP_NCRK_D339L	TTCACAAGTCGGTGTAAGATTCTTGGAGC GGCAGC	
FP_NCRK_K238E	AAAACCGCTGCGATCGAACGTCTAAATA CCCCTAA	
RP_NCRK_K238E	AGACGATCGTTCGCAGCGGTTTTACCGTC TTTGAGCT	
F0_NCRK promoter	CAGTGAAGACAAGGAGTGACAAATCTTT GGGAGTATATA	Cloning NCRK and HPCA1 by Golden gate
R0_NCRK promoter	CAGTGAAGACAACATTTGCCAGCGAGTG GAGATTAGATA	
F0_HPCA1_promoter	CAGTGAAGACAAGGAGCGGACAAACCG AGAAAGTGAAAC	
R0_HPCA1_promoter	CAGTGAAGACAAAAGACTTTGAACAAAT AGAGTTAAA	

F0_HPCA1_5'UTR	CAGTGAAGACAATCTTTATCTTCTTTTG TTAACATTATTGTTT	Cloning NCRK and HPCA1 by Golden gate
R0_HPCA1_5'UTR	CAGTGAAGACAACATTCTTCAAACCCAA AAAGAACCT	
F0_NCRK_ED	CAGTGAAGACAAAATGAAGATGAGAGTG GAAACCGCA	
R0_NCRK_ED	CAGTGAAGACAAACCTGAAAACTTGTCC TTTCTACAAATGT	
F0_HPCA1_ED1	CAGTGAAGACAAAATGAGTTCAAGAACT GGAGCCTCTTTGC	
R0_HPCA1_ED1	CAGTGAAGACAATCTGGAATTTCGCCCG TGAATTGGTTTCC	
F0_HPCA1_ED2	CAGTGAAGACAACAGAAACCCTCAGTCT CGTTAAAACGTT	
R0_HPCA1_ED2	CAGTGAAGACAATCGGAAGTAGAGTGTT CCCATGAATGGATAC	
F0_HPCA1_ED3	CAGTGAAGACAACCGATCTCCTTCTTTCT CAGGGTTGTTCAACT	
R0_HPCA1_ED3	CAGTGAAGACAAACCTGTTGCTCTTTCAG CTCTCTTCTTCT	
F0_NCRK_NS_CD	CAGTGAAGACAAAGGTCAAACCCCTTCA GTCTCTTCAGATA	
R0_NCRK_NS_CD	CAGTGAAGACAACGAAAAATATGTGCAG ATGGTTCAAGCAA	
F0_HPCA1_NS_CD1	CAGTGAAGACAAAGGTCAAAATAATCCT TTTGGTAAGACA	
R0_HPCA1_NS_CD1	CAGTGAAGACAAAATCTTCTTGTCCAATC CAATCGAATC	
F0_HPCA1_NS_CD2	CAGTGAAGACAAGATTGAAAATAGCACT TGGTTCAGGCAA	
R0_HPCA1_NS_CD2	CAGTGAAGACAAGTCACCCACAAGTTTG GAAAGACCGAAGTCGGCA	
F0_HPCA1_NS_CD3	CAGTGAAGACAATGACCCTGAGAAAACT CATGTCACAACA	
R0_HPCA1_NS_CD3	CAGTGAAGACAAATATTTGCCTCTCTCTA TCGGACTTCT	
F0_HPCA1_NS_CD4	CAGTGAAGACAAATATGTGGTGAGAGAG GTGAAAACAAAGATGAAT	

R0_HPCA1_NS_CD4	CAGTGAAGACAACGAATTGGGGCTCAAGCTTTGAAGC	
F0_NCRK_3U	CAGTGAAGACAAGCTTAAAGTAGATAGAAAAGCACAGAA	
R0_NCRK_3U:	CAGTGAAGACAAAGCGTAAATTCTAATATTAAGTAATCAAG	
F0_HPCA1_3U	CAGTGAAGACAAGCTTTTGATTATGTTTCTTTTTGTTATTT	
R0_HPCA1_3U	CAGTGAAGACAAAGCGATGGGACCTTGACCACTCTGACA	
FP-NCRK-KD-XmaI	CAGTCCCCGGGAATGAGAAAGGACAAGTTTTCT	Cloning NCRK to pGEX4T-1 vector
RP-NCRK-KD-SalI	CAGTCGTCGACTTAAATATGTGCAGATGGTTC	
FP-CML9-XmaI	CAGTCCCCGGGAATGGCGGATGCTTTCACAGAT	Cloning CML9 to pGEX4T-1 vector
RP-CML9-SalI	CAGTCGTCGACCTAATAAGAGGCAGCAATCAT	
FP-CML19-XmaI	CAGTCCCCGGGAATGGCGAATTACATGTCGGAA	Cloning CML19 to pGEX4T-1 vector
RP-CML19-SalI	CAGTCGTCGACTTAGCCGTAAGAGGTTCTCTT	
FP-CML20-XmaI	CAGTCCCCGGGAATGTCGAGTATATACAGAACT	Cloning CML20 to pGEX4T-1 vector
RP-CML20-SalI	CAGTCGTCGACCTAGTTACCACCATAAGCAGT	
FP-ROP4-XmaI	CAGTCCCCGGGAATGAGTGCTTCGAGGTTTATA	Cloning ROP4 to pGEX4T-1 vector
RP-ROP4-SalI	CAGTCGTCGACTCACAAGAACACGCAGCGGT	
FP-CML41-XmaI	CAGTCCCCGGGAATGGCAACTCAAAAAGAGAAA	Cloning CML41 to pGEX4T-1 vector
RP-CML41-SalI	CAGTCGTCGACCTAAACCGTCATCATTTGACG	
L1-HPCA1-ED-FP1	CAGTGGTCTCAAATGAGTTCAAGAACTGGAGCCTCTTTGC	Cloning NCRK/HPCA1 chimeric construct by Golden gate
L1-HPCA1-TMED-RP1	CAGTGGTCTCACGAGGCTTTTGTTTGATGACTTTG	

L1-NCRK-TMCD-FP1	CAGTGGTCTCACTCGTTATAGTTATACTACT	
L1-NCRK-NS-CD-RP1	CAGTGGTCTCACGAACCAATATGTGCAGATGGTTC	
NCRK-AttB1-FP	GGGGACAAGTTTGTACAAAAAAGCAGGCTTTATGAAGATGAGAGTGGAAACC	NCRK gateway
NCRK-AttB2-RP	GGGGACCACTTTGTACAAGAAAGCTGGGTTAATATGTGCAGATGGTTCAA	
AttB-ROP4-FP	GGGGACAAGTTTGTACAAAAAAGCAGGCTTTATGAGTGCTTCGAGGTTT	Cloning ROP4 by Gateway
AttB-ROP4-RP	GGGGACCACTTTGTACAAGAAAGCTGGGTTTCACAAGAACACGCAGCG	
GSL4-SpeI-FP	CAGTCACTAGTATGTCTCACGAAATCGTCCC	Cloning GSL4 CDS sequence with SpeI/SmaI sites
GSL4-SmaI-RP	CAGTCCCCGGGTCGATTCTTCTTCTTCCCTGCA	
mGFP6-XmaI-FP	GATCCCCGGGCTACCGGTAGAAAAAATGAGT	Cloning GFP6 and Nos terminator with SpeI/XmaI sites
mGFP6-PmeI-RP	AAACGGATCACTTCGTGTCCCAG	

Supplement Table 2. n values for experiments with unequal sample size. n is the number of images analyzed

Experiment	n	Figure
**DANS/HPTS assay**		
Col-0-Mock	17	1E
ncrk-1-Mock	20	1E
Col-0-Mock	9	2B
ncrk-1-Mock	6	2B
Col-0-H_2O_2	8	2B
ncrk-1- H_2O_2	10	2B
Col-0-Mock	18	S1D
ncrk-1-Mock	12	S1D
**Plant diameter**		
Col-0	31	1F
ncrk-1	31	1F
gsl4	34	1F
**PD index**		
RDK1-GFP	19	S1B
PDLP1-RFP	22	S1B
PDLP5-RFP	23	S1B
NCRK-GFP	46	S1B
**Interaction intensity**		
RDK1+ABI4-Mock	10	4D
NCRK+CML20-Mock	14	4D
NCRK+CML41-Mock	14	4D
RDK1+ABI4- H_2O_2	13	4D
NCRK+CML20- H_2O_2	17	4D
NCRK+CML41- H_2O_2	16	4D
**Callose staining**		
Col-0	63	1D
ncrk-1	48	1D
Col-0-Mock	34	2A
ncrk-1-Mock	22	2A
gsl4-Mock	25	2A
gsl8+/--Mock	44	2A
Col-0- H_2O_2	38	2A
ncrk-1- H_2O_2	22	2A
gsl4- H_2O_2	21	2A
gsl8+/- H_2O_2	18	2A

Col-0-Mock	12	2E
ncrk-1-Mock	17	2E
NCRK/*ncrk-1*-Mock	29	2E
Col-0- H_2O_2	12	2E
ncrk-1- H_2O_2	13	2E
NCRK/*ncrk-1*- H_2O_2	18	2E
Col-0-Mock	17	2F
ncrk-1-Mock	17	2F
$NCRK^{2CA}$/*ncrk-1*-Mock	18	2F
$NCRK^{2CA}$/*ncrk-1*-Mock	18	2F
$NCRK^{2CA}$/ncrk-1-Mock	13	2F
Col-0- H_2O_2	24	2F
ncrk-1- H_2O_2	22	2F
$NCRK^{2CA}$/*ncrk-1*- H_2O_2	15	2F
$NCRK^{3CA}$/*ncrk-1*- H_2O_2	21	2F
$NCRK^{5CA}$/*ncrk-1*- H_2O_2	12	2F
Col-0-Mock	16	3D
ncrk-1-Mock	16	3D
HPCA1/NCRK/*ncrk-1*-Mock	23	3D
Col-0- H_2O_2	13	3D
ncrk-1- H_2O_2	12	3D
HPCA1/NCRK/*ncrk-1*- H_2O_2	24	3D
Col-0-Mock	18	4E
ncrk-1-Mock	13	4E
gsl4-Mock	14	4E
ncrk gsl4-Mock	12	4E
Col-0- H_2O_2	19	4E
ncrk-1- H_2O_2	15	4E
gsl4- H_2O_2	16	4E
ncrk gsl4- H_2O_2	15	4E
Col-0-Mock	18	5C
ncrk-1-Mock	16	5C
$NCRK^{KE}$/*ncrk*-Mock	23	5C
$NCRK^{DL}$/*ncrk*-Mock	22	5C
Col-0- H_2O_2	30	5C
ncrk- H_2O_2	17	5C
$NCRK^{KE}$/*ncrk-1*- H_2O_2	21	5C
$NCRK^{DL}$/*ncrk-1*- H_2O_2	20	5C
Col-0-Mock	18	5D
Col-0-5 µM MV	18	5D
ncrk-0-Mock	18	5D

ncrk-5 μM MV	18	5D
Col-0-Mock	18	5E
ncrk-1-Mock	22	5E
Col-0-Wounding	19	5E
ncrk-1-Wounding	20	5E
Col-0-flg22	26	5G
ncrk-1-flg22	24	5G
Col-0	15	S2C
ncrk-1	16	S2C
Col-0	17	S2E
ncrk-1	19	S2E
ncrk-2	20	S2E
Col-0-Mock	15	S6
Col-0-0.1 μM MV	13	S6
Col-0-1 μM MV	11	S6
Col-0-5 μM MV	15	S6
